# Genomic landscapes by multiregion sequencing combined with circulation tumor DNA detection contribute to molecular diagnosis in glioblastomas

**DOI:** 10.18632/aging.102526

**Published:** 2019-12-10

**Authors:** Chao Yang, Yanli Tan, Shouwei Li, Junhu Zhou, Qixue Wang, Yunfei Wang, Yingbin Xie, Luyue Chen, Jie Li, Chuan Fang, Chunsheng Kang

**Affiliations:** 1 Department of Neurosurgery, Tianjin Medical University General Hospital, Tianjin, China; 2 Laboratory of Neuro-Oncology, Tianjin Neurological Institute, Department of Neurosurgery, Tianjin Medical University General Hospital and Key Laboratory of Neurotrauma, Variation, and Regeneration, Ministry of Education and Tianjin Municipal Government, Tianjin, China; 3 Department of Pathology, Affiliated Hospital of Hebei University, Baoding, China; 4 Department of Pathology, Hebei University Medical College, Baoding, China; 5 Department of Neurosurgery, Beijing Sanbo Brain Hospital, Capital Medical University, Beijing, China; 6 Department of Neurosurgery, Zhongshan Hospital Xiamen University, Xiamen, Fujian, China; 7 ProteinT Biotechnologies, Co. Ltd., Tianjin, China; 8 Department of Neurosurgery, Affiliated Hospital of Hebei University, Baoding, China; 9 Affiliated Cancer Hospital and Institute of Guangzhou Medical University, Guangzhou, China

**Keywords:** glioblastoma, intratumor heterogeneity, exon sequencing, ctDNA sequencing

## Abstract

Glioblastoma is a highly aggressive brain malignancy with a poor prognosis. Its high intratumor heterogeneity contributes to therapeutic resistance, tumor progression and recurrence. We sequenced 31 loci in 11 patients with glioblastoma (including one patient with samples available from the primary and recurrent tumors) to determine the genetic basis and intratumor heterogeneity of glioblastoma. By analyzing the somatic mutations, known driver genes were identified, including EGFR, PTEN and TP53, and the MUC16 gene exhibited the highest mutation rate in the samples examined. Through an evolutionary analysis of the sequencing results, the EGFR p.L861Q mutation was determined to play a role in the progression from the primary tumor to a relapsing tumor in one patient. We analyzed 1403 genes in blood-derived ctDNA that were previously revealed to play a role in tumorigenesis and the progression of cancer. Somatic mutations identified through ctDNA sequencing that match the results of multipoint exon sequencing in tumor tissues were detected, such as EGFR p.L861Q. These findings provide new insights into the intratumor heterogeneity and evolution of glioblastoma. In addition, ctDNA detection in blood samples represents a convenient method to dynamically identify the genetic changes and new therapeutic targets during the treatment of glioblastoma.

## INTRODUCTION

Glioma is the most common primary intracranial tumor in adults, among which glioblastoma multiforme (GBM) has the highest degree of malignancy and a poor prognosis, with average survival rate of less than 15 months and a 5-year survival rate of less than 10% [1]. Currently, glioma is primarily treated with surgical resection, radiation and chemotherapy. The concurrent addition of temozolomide (TMZ) to radiation as a chemotherapy adjuvant modestly improves survival among young patients with a good performance status and has become the standard of care [2]. Despite the benefits of TMZ, tumors invariably recur, leading to a fatal outcome. Therefore, a more in-depth understanding of the occurrence and progression of glioma will be beneficial for the development of personalized treatment.

Extensive genetic diversity in GBM results in resistance to standardized treatment and a poor prognosis. Through a recent exploration at the genetic level, a new strategy for obtaining a better understanding of and improving GBM treatment was discovered and proposed [1]. In particular, individualized targeted therapy is selected for individual tumor mutations [3, 4]. Although this approach seeks to maximize the drug response and patient survival, the intratumor heterogeneity of GBM poses significant challenges [5–7]. Specifically, each tumor contains multiple clones with different genetic alterations, which will require strategies designed to therapeutically target multiple molecules [5, 8]. The detection of a single tumor locus may not accurately reflect the genetic characteristics of other tumor regions, rendering the traditional biopsy prone to errors and posing a significant challenge in cancer medicine [9].

Tumor heterogeneity has been used to describe various forms of tumor variability, including variations in the intertumoral mutation pattern, variations in intratumor histology and intratumor mutational polyclonality [10]. Spontaneous somatic cell mutations combined with the microenvironment for the evolutionary selection of tumor subclones will promote the growth of single cancer cells into complex and heterogeneous tumor masses [11]. During the evolution of clones, new mutations become more frequent as tumors progress, increasing the difficulty of treating these tumors. The poor prognosis of patients often indicates the progression of tumor heterogeneity [12–14].

Based on accumulating evidence, GBM can be further classified at the genomic level to reveal the evolution of tumors [5]. In addition, tumor fragments from the same patient can be divided into different GBM subtypes [6]. In the present study, subclones were detected in patients with GBM prior to treatment and new subclones appeared in the same patients after standardized treatment. We also describe a subset of tumor-associated genetic changes in blood-derived ctDNA.

## RESULTS

### Known driver gene mutations and significantly mutated genes (SMGs) in GBM samples

All point mutations were expressed in the following 6 forms: C>A(G>T), C>G(G>C), C>T(G>A), T>A(A>T), T>C(A>G), and T>G(A >C). Tumor samples and point mutation types were clustered according to the number of point mutations (Supplementary Figure 1A). As expected, we detected a point mutation variation in samples collected at different loci of the same original tumor, but in the patients with recurrent tumors (NO. 05-recurrent), the mutation variation was less than the original sample (NO. 05-primary) (Supplementary Figure 1A). The overall mutation pattern of GBM was dominated by C>T and G>A (Supplementary Figure 1A), particularly in recurrent samples (derived from NO. 05-recurrent).

**Figure 1:**
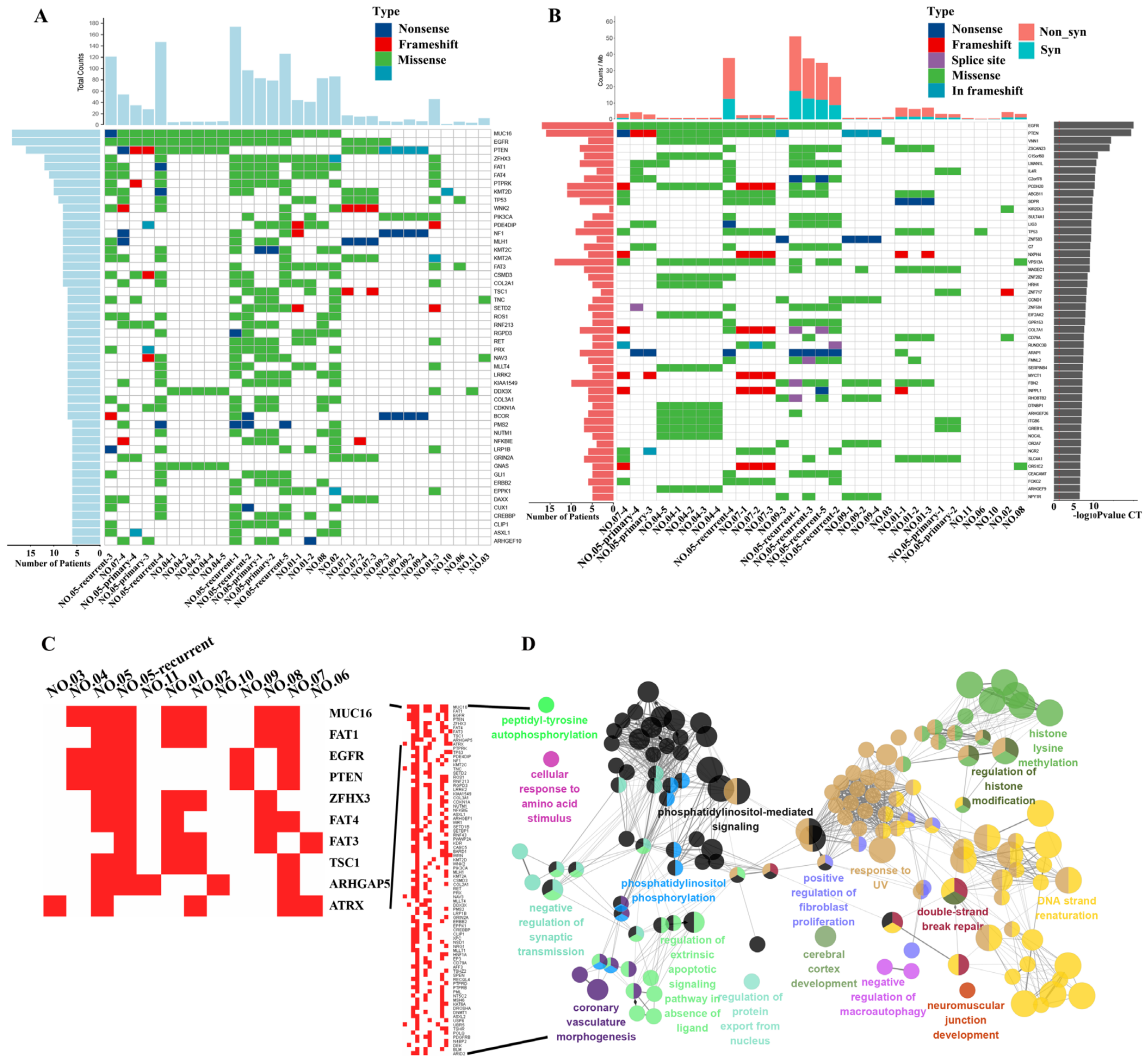
**Driver gene mutation analysis and significantly mutated genes (SMGs) in GBM.** (**A**) The top 50 driver genes with the highest mutation frequencies were selected for inclusion in the heat maps. The abscissa lists the sample names and the ordinate lists the gene names. The left graph shows the number of mutant samples and the top graph shows the number of mutated genes in each sample. (**B**) The graph on the right shows the log10 P-value of each gene mutation. The heat map (middle panel) presents gene mutations in GBM samples. The graph on the left shows the mutation frequency in the GBM samples examined. The mutant load is shown on the top of the heat map. (**C**) Mutation heatmap. (**D**) GO analysis.

We next identified the driver gene mutations in these GBM samples using the CGC513 (https://cancer.sanger.ac.uk/census), Bert Vogelstein125 [15] and SMG127 [16] driver mutation databases for comparison. We subsequently selected the top 50 driver gene mutations for mapping and observed higher mutation frequencies for MUC16 (a 19/31 ratio), EGFR (a 19/31 ratio) and PTEN (a 16/31 ratio) (Figure 1A). The IDH1 mutation was detected in two patients (NO. 03 and NO. 05-recurrent) at 18% (not shown in the figure). The MUC16 gene, also called CA125, was recently shown to play a pivotal role in promoting ovarian cancer growth and metastasis [17] and is associated with a higher tumor mutation load (TML), better survival outcomes and better immune response in patients with gastric cancer [18].

We also identified 55,992 SNVs from our exon sequencing of 31 GBM loci. High-frequency mutations in GBM were analyzed using MuSiC software [19] and the convolution test (CT), Fisher’s test (Fisher’s combined P-value test, FCPT), and the likelihood ratio test (LRT). If the false discovery rate (FDR) ≤ 0.2 in two of three tests, the gene was considered a high-frequency mutation. Sixty-seven SMGs, including EGFR, PTEN, VNN1, and ZSCAN23, were identified (the top 50 are shown in Figure 1B). We analyzed our SMG data with the PathScan module of MuSiC [19] software to perform a pathway enrichment analysis and obtain insights into the genetic alterations in canonical signaling pathways. The significantly enriched pathways of high-frequency mutated genes were revealed and included focal adhesion, axon guidance, NGF signaling and the cAMP signaling pathway, among others (Supplementary Figure 1B). In addition, heatmaps of SMGs in tumor-related pathways were used to observe the variations in the mutation frequencies (Supplementary Figure 2). Again, we observed intratumor heterogeneity in the mutations in genes involved in these pathways associated with tumor formation and progression.

### MUC16, the gene with the highest mutation frequency, indicates a good prognosis

We counted the gene mutations obtained from multipoint sequencing in 11 patients, and the 10 genes with the highest mutation frequencies were MUC16, FAT1, EGFR, PTEN, ZFHX3, FAT4, FAT3, TSC1, ARHGAP5 and ATRX (Figure 1C). FAT1, FAT3 and FAT4 are cadherin family members, and this family of molecules is likely to be important in mammalian developmental processes and cell communication [20]. The loss of FAT1 also contributes to the mechanism of resistance mediated by CDK4/6i in ER^+^ breast cancer [21]. EGFR, PTEN and ATRX are commonly mutated genes in glioma [1]. Multipoint sequencing can identify new high-frequency mutated genes in GBM. By analyzing the GO biological processes of genes that were mutated in more than three patients, we mainly observed enrichment in double-strand break repair, histone modification and phosphatidylinositol-mediated signaling, among other processes (Figure 1D).

**Figure 2:**
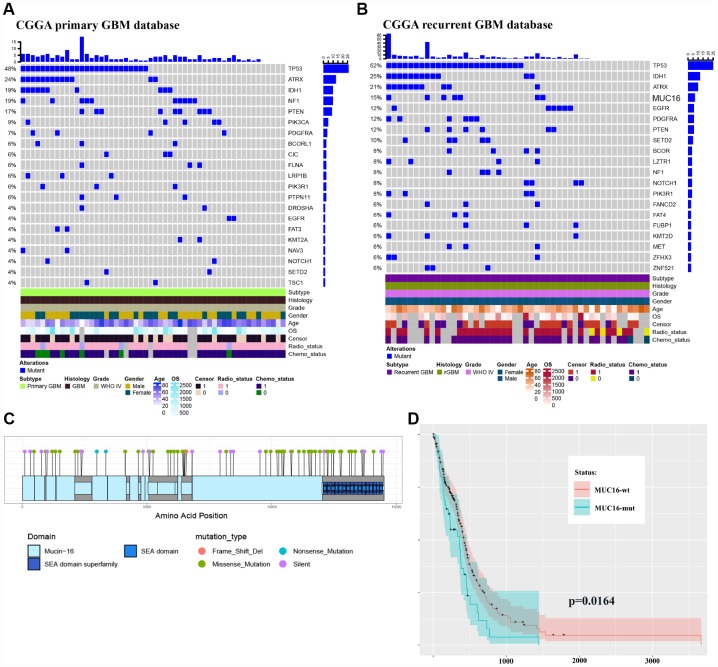
**Multipoint sequencing provides a new map of mutations.** (**A**–**B**) Genes that were mutated in the patient cohort were detected in CGGA primary and recurrent GBM WES databases. (**C**) MUC16 mutations in the TCGA database. (**D**) Survival curves of patients carrying wild type and mutant MUC16.

Few mutations were identified in MUC16 in primary GBM samples, but a 15 percent mutation frequency was observed in recurrent GBM (Figure 2A–2B) in the CGGA (Chinese Glioma Genome Atlas) GBM database, validating our results. This finding indicates the importance of MUC16 in GBM recurrence. The most frequently mutated gene, MUC16, in our sequencing data also contains multiple mutation sites in the gene mutation data provided by TCGA (Figure 2C). By analyzing the TCGA clinical data, the overall survival curve showed that patients with GBM carrying a mutation in the MUC16 gene had a poor survival outcome (Figure 2D). Based on these results, multipoint sampling is a better indicator of underlying tumor progression.

### Analysis of subclones and the evolution of primary and recurrent GBM samples from the same patient

**Figure 3:**
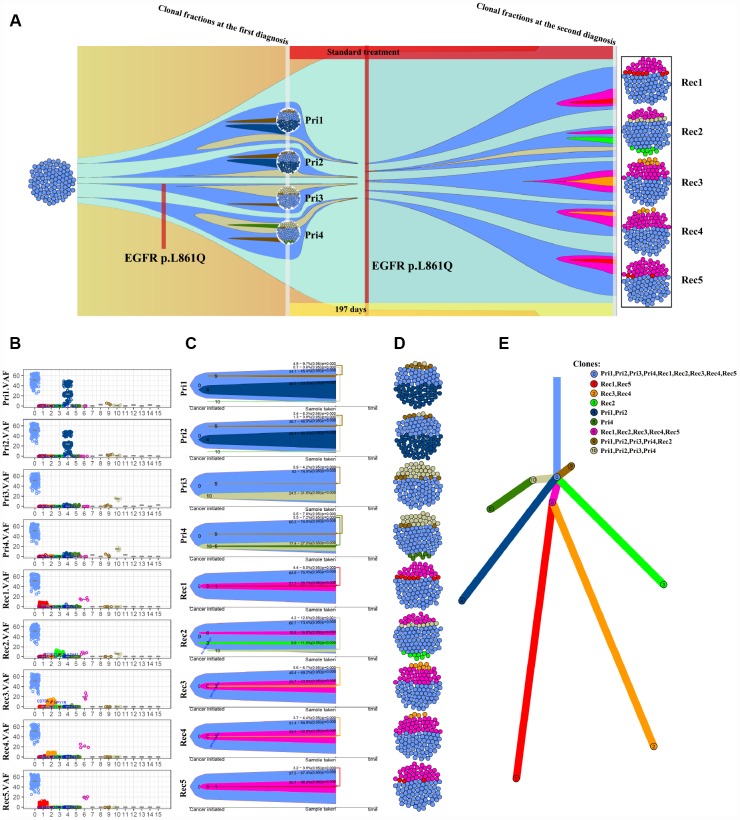
**Analysis of the subclones and evolution of primary and recurrent GBM samples from patient NO. 05.** (**A**) Four intra-tumor loci from the primary tumor and 5 intra-tumor loci from the recurrent tumor from the same patient were sequenced and analyzed. (**B**) VAF distribution of different subclone types at each intratumor locus. (**C**) A clonal evolution map of each intratumor locus. (**D**) Clonal structure distribution of each intratumor locus. (**E**) A tumorigenic chart of intratumor loci.

We next outperformed a subclone analysis of primary and recurrent GBM samples from patient NO. 05 with the aim of exploring the genetic evolution of GBM. Multiple subclones were detected in each site of primary and recurrent tumor samples from patient NO. 05, but the mutated genes and frequency between tumor sites differed (Figure 3A and 3B). Clone 0 was present at a higher proportion in all sites, but clone 4 was present at a higher proportion in pri1 (NO. 05-primary 1) and pri2 (NO. 05-primary 2). Notably, clone 6 accounted for a higher VAF in all recurrent samples (Figure 3B–3D), suggesting that the gene mutations present in clone 6 play an important role in GBM recurrence. Further analysis using Pyclone CCF package ClonEvol [22] revealed the evolutionary relationship between the primary and recurrent GBM tumors in patient NO. 05 (Figure 3E). An analysis of the subclones and evolutionary relationship in patients NO. 05 and NO. 04 (Supplementary Figure 3) indicated that different branches appeared during GBM progression, and a complex pattern of intratumor heterogeneity gradually formed in these tumors. H&E (Supplementary Figure 4) and immunofluorescence staining (Supplementary Figure 5) of the tumors from patient NO. 05 also revealed significant intratumor differences.

### The ctDNA in the blood predicts the overall genetic information of the GBM

**Figure 4:**
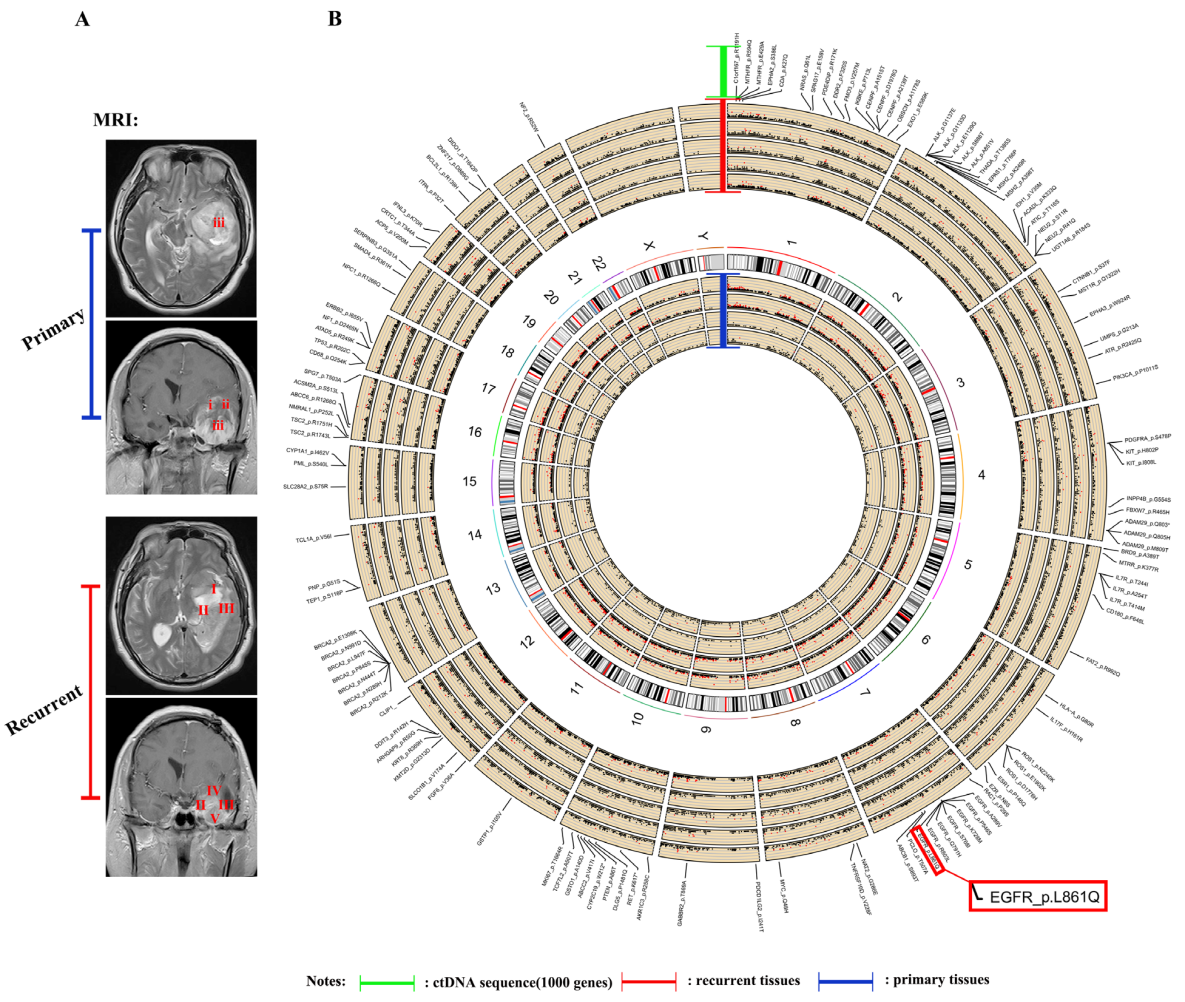
**Sequencing and analysis of the ctDNA.** (**A**) Magnetic resonance images show the different locations of tissue samples derived from patient NO. 05. (**B**) SNP density circos map of the primary and recurrent GBM samples from patient NO. 05. The first circle represents sample (**i**), the second circle represents sample (**ii**), the third circle represents sample (**iii**), the fourth circle represents the repeat of sample (**iii**), the fifth circle represents chromosomes, the sixth circle represents sample (**I**), the seventh circle represents sample (**II**), the eighth circle represents sample (**III**), the ninth circle represents sample (**IV**), the tenth circle represents sample (**V**), and the eleventh circle represents gene mutations detected using ctDNA testing.

**Figure 5:**
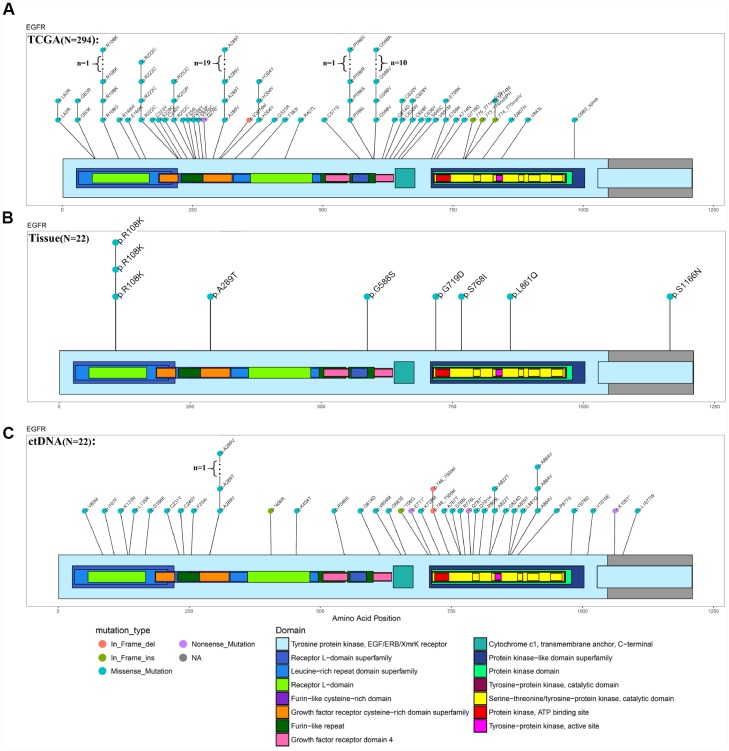
**Comparison of EGFR mutations identified using ctDNA and tumor DNA sequencing.** (**A**–**C**) Mutation hotspots of EGFR mutations present in the TCGA database, patient tissue WES data and ctDNA testing results.

The ctDNA in the blood carries the genetic information of the tumor and has become an indicator of the occurrence and progression of tumors following a liquid biopsy [23–25]. Because of the limited amount of ctDNA in the blood, we were unable to detect all genetic mutations identified in the tumors. In this study, we selected 1,403 common genes, which are known to be involved in tumor progression, and detected their common mutation sites for evaluation in ctDNA. Importantly, the EGFR p.L861Q mutation in the ctDNA of patient NO. 05 was detected in all recurrent samples but only in some of the original samples (Figure 4). By comparing the ctDNA sequencing data with tumor tissue exon sequencing results from all 11 patients with GBM and the TCGA gene mutation database, ctDNA sequencing detected common gene mutations in GBM tumors (Figures 5A–5C, Supplementary Figure 6 and Supplementary Figure 7) and obtained additional gene mutation information that failed to be detected in tumor tissue samples (Figure 5B and 5C).

## DISCUSSION

GBM shows obvious molecular heterogeneity and invasive behavior [5, 7, 10, 26]. The heterogeneity of a tumor refers to the genetic changes in its subpopulations of cells after multiple division sand proliferation during tumor growth [5, 6, 11, 27–30]. Following the development of new detection technology, the most recent data, including next-generation sequencing data, support the clonal evolution model as the main theoretical basis of heterogeneity [30–32]. By sequencing 31 intratumor loci and ctDNAs from 11 patients with primary and recurrent GBM, we observed high intratumor heterogeneity at the levels of both somatic gene mutations and chromosomal copy numbers in the present study. The intratumor heterogeneity of GBM has been suggested to contribute to therapeutic resistance [33], tumor progression and recurrence [34]. Interestingly, even recurrent GBM presented a high degree of genetic heterogeneity in the present study. Moreover, the subclone analysis revealed different signatures of intratumor heterogeneity between primary and recurrent GBM (Figure 3A–3C). For instance, clone 6 was only present in recurrent tumors (Figure 3B), suggesting that it may represent a causal factor of recurrent GBM that is more refractory to treatment than the primary tumor. These results suggest the selective evolution of the tumor subclone structure after treatment (Figure 3A). Further investigations of the underlying detailed molecular changes and driving force of the heterogeneity in recurrent GBM might substantially improve the therapeutic efficacy and prognosis of GBM.

Additional treatment strategies for GBM are urgently needed. Precision medicine has been a major focus in the field, with the goal of matching specific tumor mutations with potential therapeutic drugs to provide individualized treatment options [35, 36]. An increasing number of studies have identified a number of driver gene mutations, including TP53, PTEN, EGFR, PIK3CA, ATRX, IDH1, PIK3R1, and PDGFRA, MSH6 and PRDM2, in GBM [1, 37, 38]. Consistent with these findings, frequent mutations in these known driver genes were detected in our GBM samples (Figure 1A and 1C). In the present study, MUC16 was the most frequently mutated known driver gene in the GBM tissue samples sequenced (Figure 1A). Studies of MUC16 in other tumor types have indicated that MUC16 is a critical therapeutic target [17, 18]. For instance, an antibody (DUC5754A) against MUC16 has been advanced to a phase 1 clinical trial for ovarian cancer [39]. In addition, we detected the EGFR p.L861Q mutation in all intratumor loci in recurrent tumors but in a few loci in primary GBM tumors from patient NO. 05, suggesting that the EGFR p.L861Q mutation potentially represents a marker for predicting recurrence.

Based on accumulating evidence, ctDNA, which is released into the blood after the apoptosis of tumor cells, has potential as a tumor biomarker [40–43]. Moreover, ctDNA provides a comprehensive picture of the tumor genome, as it reflects the DNA released from multiple tumor regions [44–47]. Recently, ctDNA has been detected in the cerebrospinal fluid (CSF) from patients with brain malignancies [44]. Compared to ctDNA testing, single-site biopsy or several reliable biopsies are needed to obtain the same amount of genetic information [44–47]. However, ctDNA also has some substantial limitations, such as the generally very low level of ctDNA in plasma, resulting in a small range of genes that are able to be detected using sequencing [48]. In the present study, we selected 1,403 genes involved in tumorigenesis and progression for detection and compared them with the sequencing results obtained from intratumor loci. Importantly, the information obtained from the analysis was highly consistent with the findings from the sequencing of intratumor loci (Figures 4 and 5). Thus, blood-derived ctDNA can be used as a liquid biopsy to help diagnose and monitor GBM.

Our study has provided a better understanding of intratumor heterogeneity, disease progression and recurrence in patients with GBM. Our analysis of the temporal sequence of mutations and chromosomal copy number revealed genetic forces acting on the cancer genome and provided new insights into the patterns and dynamics of tumor evolution. Further investigations will provide more detailed descriptions of the mechanisms of disease relapse and new therapeutic strategies for this disease.

## MATERIALS AND METHODS

### Blood specimen collection and preservation

Streck noninvasive blood vessel collection tubes (218962, 10 mL, cell-free DNA BCT) were used to collect venous blood prior to surgery. The supernatant and cell pellet were separately preserved after centrifugation. The first centrifugation was conducted at 1600 × g for 10 minutes at 4°C. The blood cell pellet was collected after centrifugation and stored at -80°C. The upper plasma fraction was subjected to a second centrifugation step (4°C, 16000 × g, 10 minutes). The upper plasma fraction produced after second centrifugation step was collected and stored at -80°C.

### Clinical samples

Our study was approved by the Ethics Committee of the Affiliated Hospital of Hebei University (HDFY-KL-LL-2018-17). All tissues were stored in liquid nitrogen for whole exome sequencing (WES). Clinical data for the tumor samples used in this study are listed in the supplementary table (Supplementary Table 1).

### DNA sequencing

Qualified tissue and blood cell DNA samples were randomly disrupted into 150- to 220-bp fragments by Covaris, and then the Agilent SureSelect Human All Exon V6/V7 kit was used for library construction and capture. The captured library was subjected to whole-exome sequencing to identify somatic mutations, including single nucleotide variants (SNVs), insertions or deletions (INDELs), and copy number alterations (CNAs). A 100X target coverage of coding bases in the exome was achieved for all samples.

The ctDNA was extracted from the upper centrifugal plasma of patients. A captured ctDNA library was then constructed. The purified library was sequenced using a NovaSeq 6000 sequencer. The ctDNA sequencing method was based on next-generation high-throughput sequencing (NGS) and designed to detect a variety of solid tumor- and drug-related gene mutations. The panel design adopted Roche NimbleGen SeqCap probe design technology and covered hot spots, drug-related mutations and key areas of 1403 genes to ensure the comprehensive detection of hot spots and rare and unknown mutation sites to the maximum extent and to ensure that valuable mutation information for patients was not ignored.

### Bioinformatics analyses and statistical analyses

The caner genome atlas (TCGA) somatic mutation (SNPs and small INDELs) data used in this study were retrieved from the following website: https://xena.ucsc.edu/welcome-to-ucsc-xena/. CGGA [49] WES sequencing results were analyzed using the website: http://cgga.org.cn/index.jsp. GO and survival analyses were performed using the R package Clusterprofiler [12] and survminer v0.2.1 (https://www.rdocumentation.org/packages/survminer). The mutation hotspot graphic was generated using the R package GenVisR [13]. The genome circos map was produced using the R package Rcircos [14] (https://www.rdocumentation.org/packages/RCircos). The nonredundant biological terms for large clusters of genes in a functionally grouped network were visualized using the Cytoscape plug-in clueGO [50].

### Somatic SNV test

SNV is defined as a single nucleotide variant, which refers to the variation caused by the replacement of a single nucleotide in the genome. We used MuTect [51] software to search for somatic SNVs and annotated the results with Annovar software.

### Somatic INDELs/SNVs/SVs

The Somatic INDEL sites were detected using Strelka [52] and annotated using Annovar software. We used Control-FREEC [53] and lumpy [54] to detect somatic CNVs and SVs in paired samples of tumor and normal tissues.

### Somatic mutations

The mutation spectrum and mutation signature were analyzed. All point mutations were reported in the following 6 forms: C>A(G>T), C>G(G>C), C>T(G>A), T>A(A>T), T>C(A>G), and T>G(A>C). Tumor samples and point mutation types were clustered according to the number of point mutations. The preference of point mutation types and the degree of similarity of each tumor sample were studied. Mutation characteristics were analyzed to extract the mutation features of somatic point mutations based on the number of 96 point mutations in various tumor samples and using non-negative Matrix Factorization (NMF) [55]. Each mutation feature reflects the physical, chemical or biological process of a cancer somatic mutation. The COSMIC web site lists more than 30 known mutations.

### Driver mutations

We compared somatic cell variations with known driver genes and screened known driver genes in these tumor samples. The driver genes used for comparison were derived from the following sites:

1. CGC513: the driver genes listed in the Cancer Gene Census (https://cancer.sanger.ac.uk/census);
2. Bert Vogelstein125: 125 mut-driver genes in the paper by Bert Vogelstein and colleagues [15];
3. SMG127: significant mutated genes identified in pan-cancer data [16].

### Significantly mutated genes (SMGs)

High frequency mutations in tumors were analyzed using MuSiC [19] software. MuSiC sets somatic cell mutations in all tumor samples as the background, performs statistical tests on various mutation types in genes, and detects genes with a significantly higher mutation frequency than the background mutation rate. MuSiC software performs the SMG test using three methods, including a convolution test (CT), Fisher’s test (Fisher’s combined P-value test, FCPT), and likelihood ratio test (LRT). If FDR ≤ 0.2 in two inspections, the gene will be categorized as a high-frequency mutation. PathScan [56] (a module of MuSic) was used to analyze high-frequency mutated genes. The metabolic pathway databases used for enrichment were KEGG, PID and Reactome.

### Evolutionary tree analysis

According to the mutation frequency identified in somatic sites as the Vaf (variant allele frequency) combined with copy number variations, Pyclone software was used to calculate the proportion of mutant cells in the CCF (cancer cell fraction) of tumor and studied the clone structures. ClonEvol [22] software was employed to analyze the evolutionary relationship between tumor samples.

### H&E staining, immunofluorescence staining, and confocal imaging

Paraffin-embedded tissue sections were used for H&E staining. For immunofluorescence staining, the sections were incubated with primary antibodies (1:100 dilution) (p-EGFR and POLK, from CST Cell Signaling Technology) overnight at 4°C, followed by a 1-h incubation with a fluorescently labeled secondary antibody (1:100 dilution) at 37°C. Images were obtained with a confocal microscope (Olympus FluoView 1200 system). All confocal scanning parameters were maintained at constant values between samples, and the images were minimally processed to maintain the integrity of the data.

## Supplementary Material

Supplementary Figures

Supplementary Table 1
