# Supplementary material for: Genomic landscapes by multiregion sequencing combined with circulation tumor DNA detection contribute to molecular diagnosis in glioblastomas
Source: Aging (Albany NY). 2019 Dec 10;11(23):11224–43. doi: 10.18632/aging.102526 (PMC6932900; doi:10.18632/aging.102526)
Supplement: Supplementary Figures [file aging-11-102526-s002..pdf]

SUPPLEMENTARY FIGURES

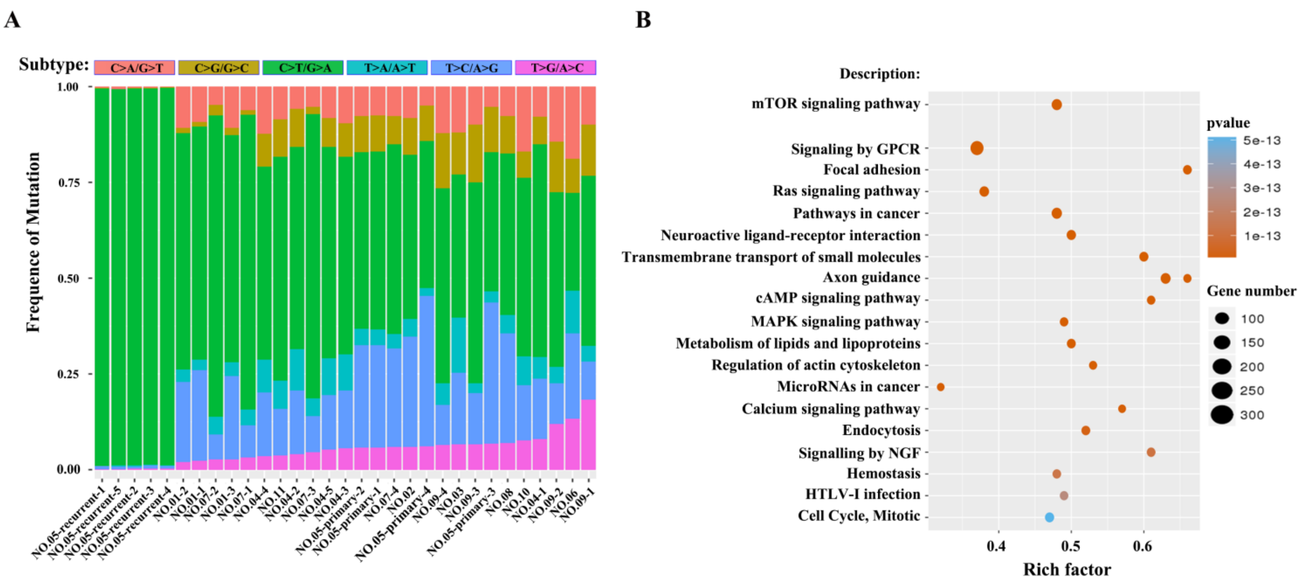

Supplementary Figure 1. (**A**) The abscissa indicates the sample name, the ordinate indicates the proportion of each mutation type in the sample, and different colors represent different SNV mutation types. (**B**) Pathway analysis of SMGs. The ordinate provides the pathway description, the abscissa lists the gene detection rate in each pathway, the size of the dot represents the number of genes in each pathway and the color of the dot represents the range of p values.

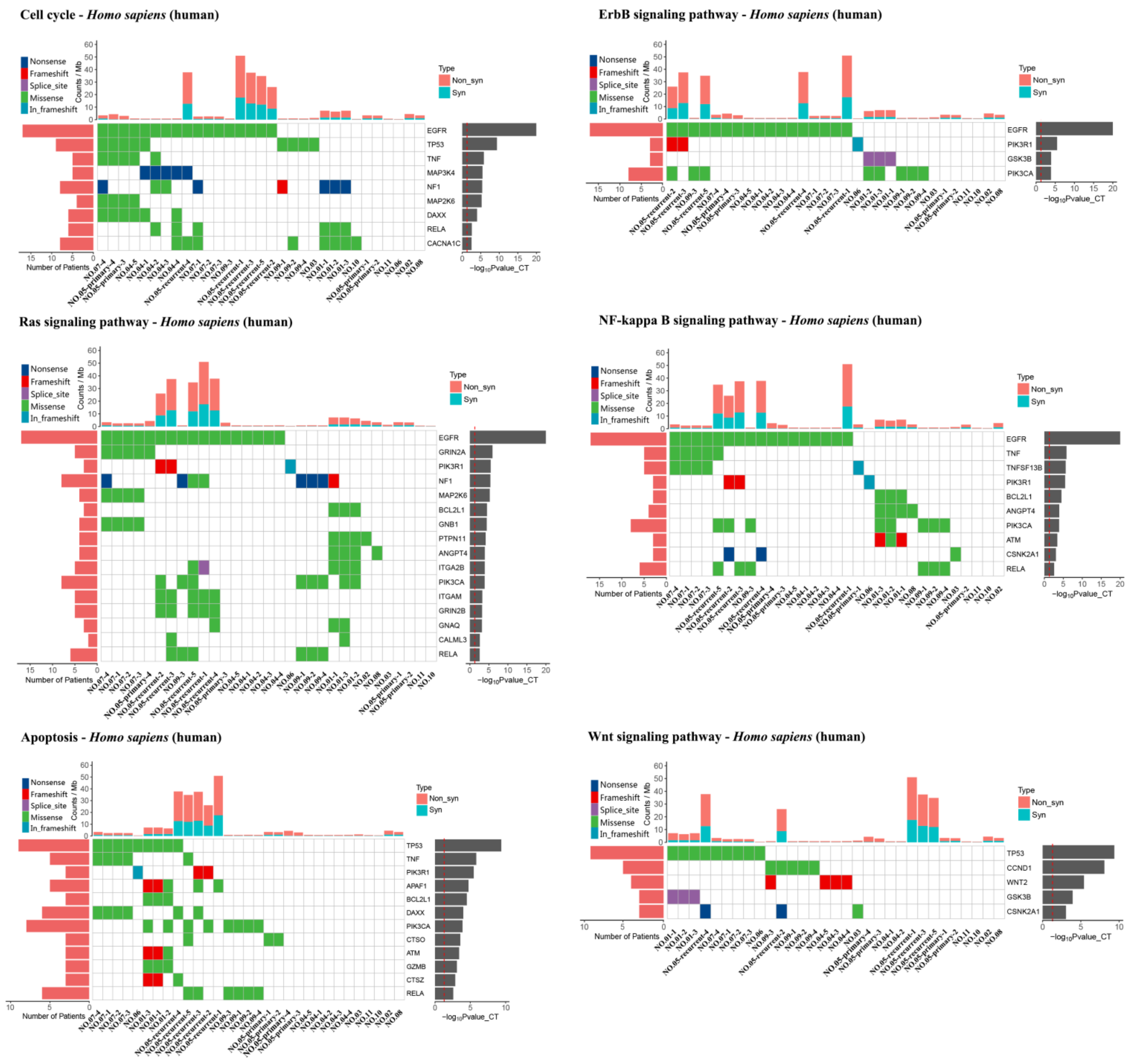

Supplementary Figure 2. Significantly mutated genes (SMGs) involved in the cell cycle, ErbB signaling, RAS signaling, NF-kappa signaling, apoptosis and the wnt signaling pathway. The graph on the right presents the log10 P-value of each gene mutation. The heat map (middle panel) presents gene mutations in GBM samples. The graph on the left shows the mutation frequency in the GBM samples examined. The mutant load is shown on top of the heat map.

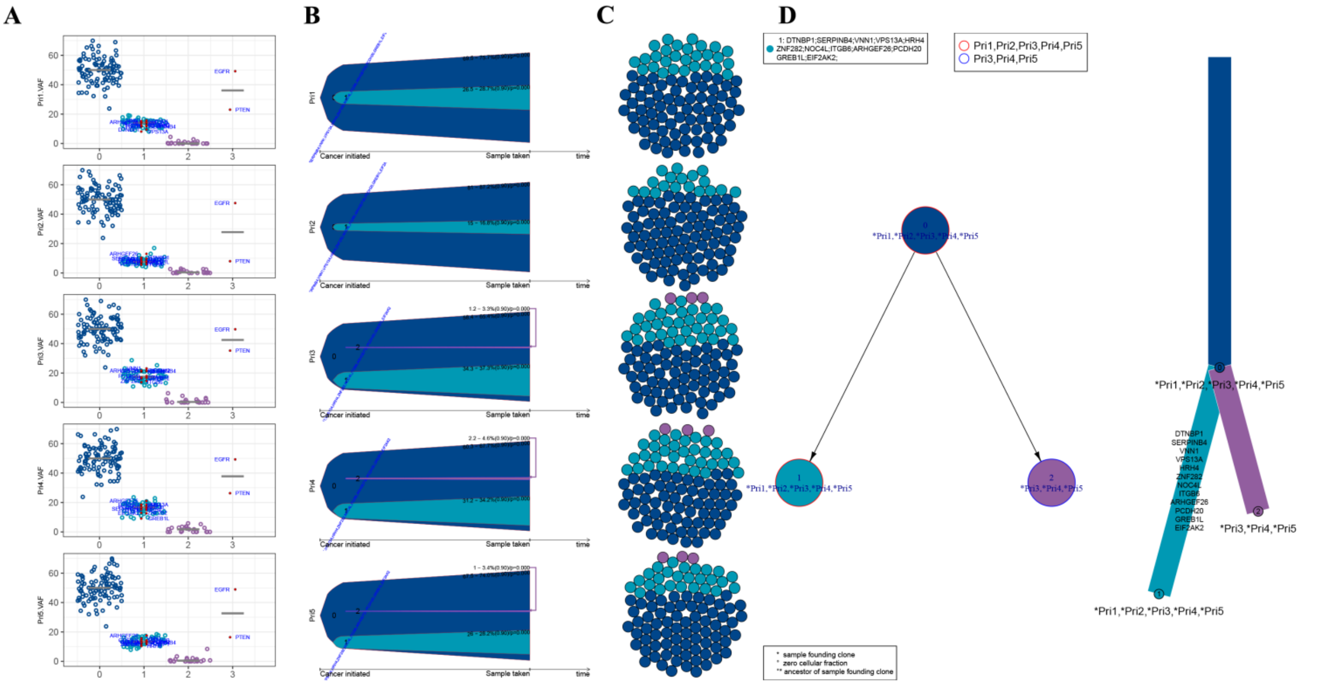

Supplementary Figure 3. Analysis of the subclones and evolution of GBM samples from patient NO. 04.

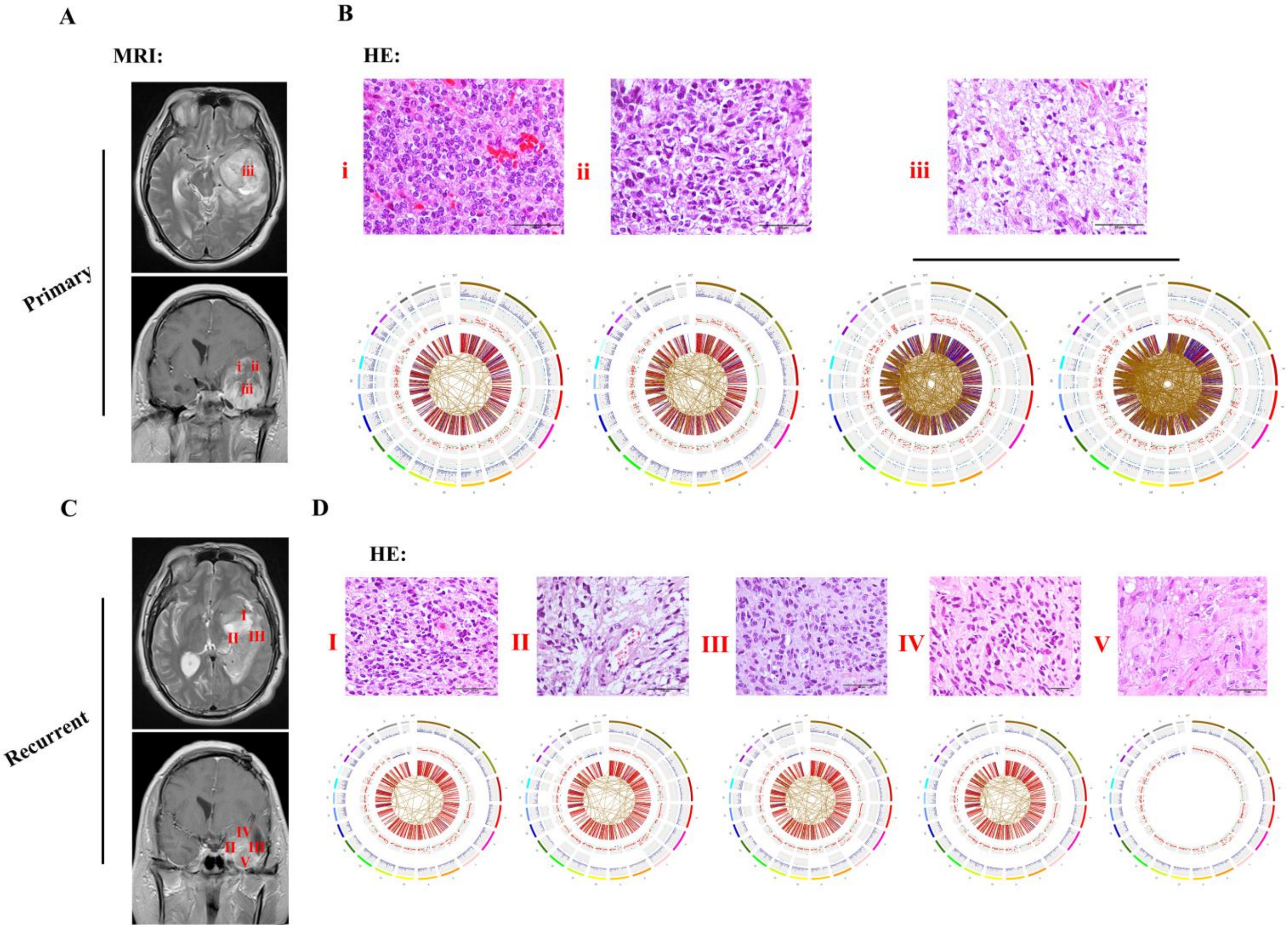

Supplementary Figure 4. (**A**) Magnetic resonance images of the primary glioblastoma tumor from patient NO. 05. (**i**, **ii**, and **iii**) indicate the locations of the multipoint samples. (**B**) HE staining and genome mutation circos plots of primary multipoint samples from patient NO. 05. The first circle indicates chromosomes. The dark purple dots in the second circle represent the density of SNPs, The dark blue points in the third circle denote the density of INDELs. The fourth circle presents the CNV results, where red indicates an increased copy number, blue indicates a decreased copy number, and green indicates a normal copy number. The fifth circle presents the SV results. Due to the large amount of data, only SV data from exons and splice sites are displayed: CTX (brown), ITX (blue), INS (orange), DEL (dark red), DUP (light purple) and INV (green). (**C**) Magnetic resonance images of the recurrent tumors from patient NO. 05. (**I**-**V**) indicate the locations of the multi-point samples. (**D**) HE staining and genome mutation circos plots of recurrent multipoint samples from patient NO. 05.

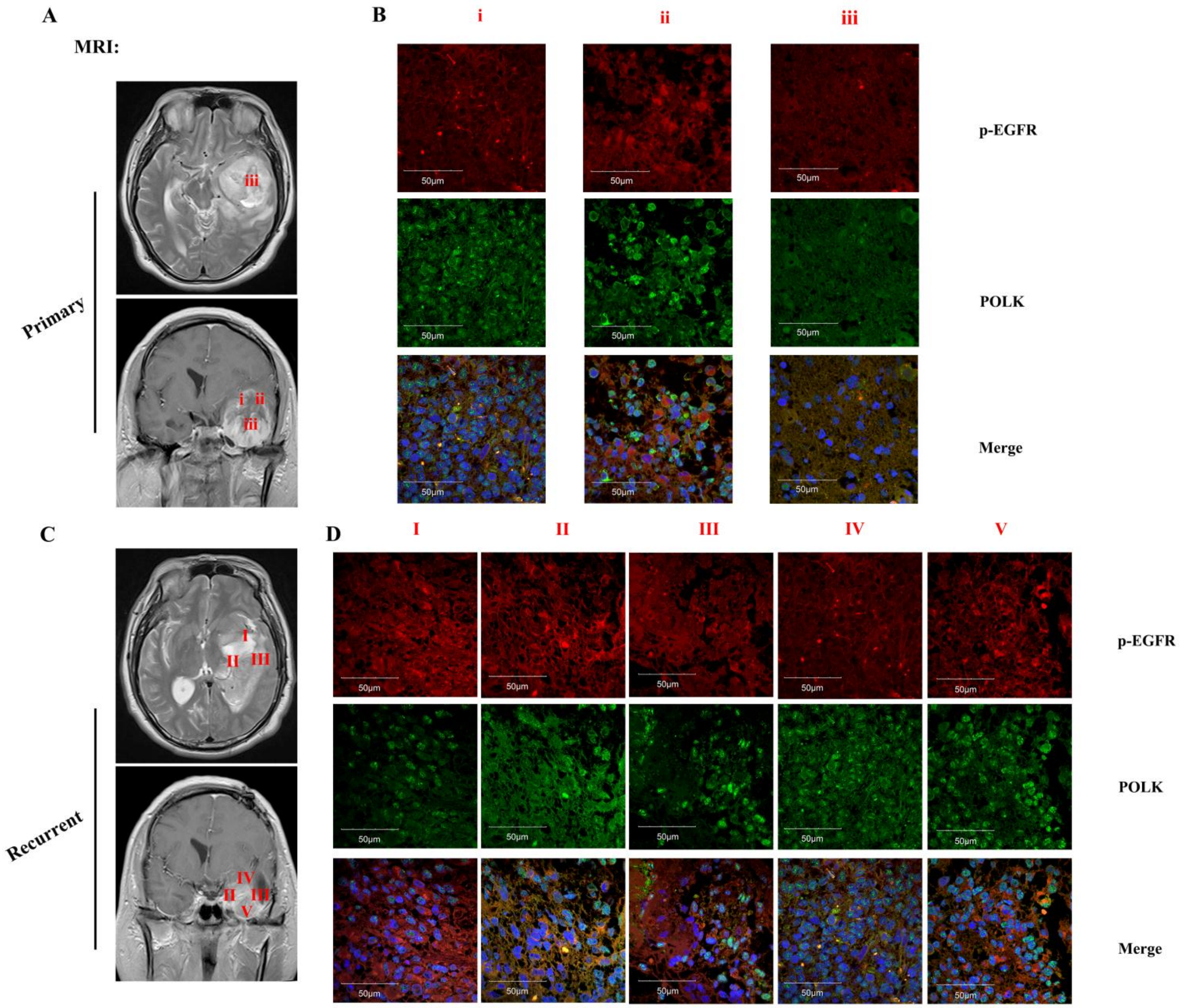

Supplementary Figure 5. (**A**) Magnetic resonance images of the primary glioblastoma tumors from patient NO. 05. (**i**–**iii**) indicate the locations of the multi-point samples. (**B**) Immunofluorescence staining of primary multipoint samples from patient NO. 05. (**C**) Magnetic resonance images of the recurrent tumors from patient NO. 05. (**I**–**V**) indicate the locations of the multi-point samples. (**D**) Immunofluorescence staining of recurrent multipoint samples from patient NO. 05.

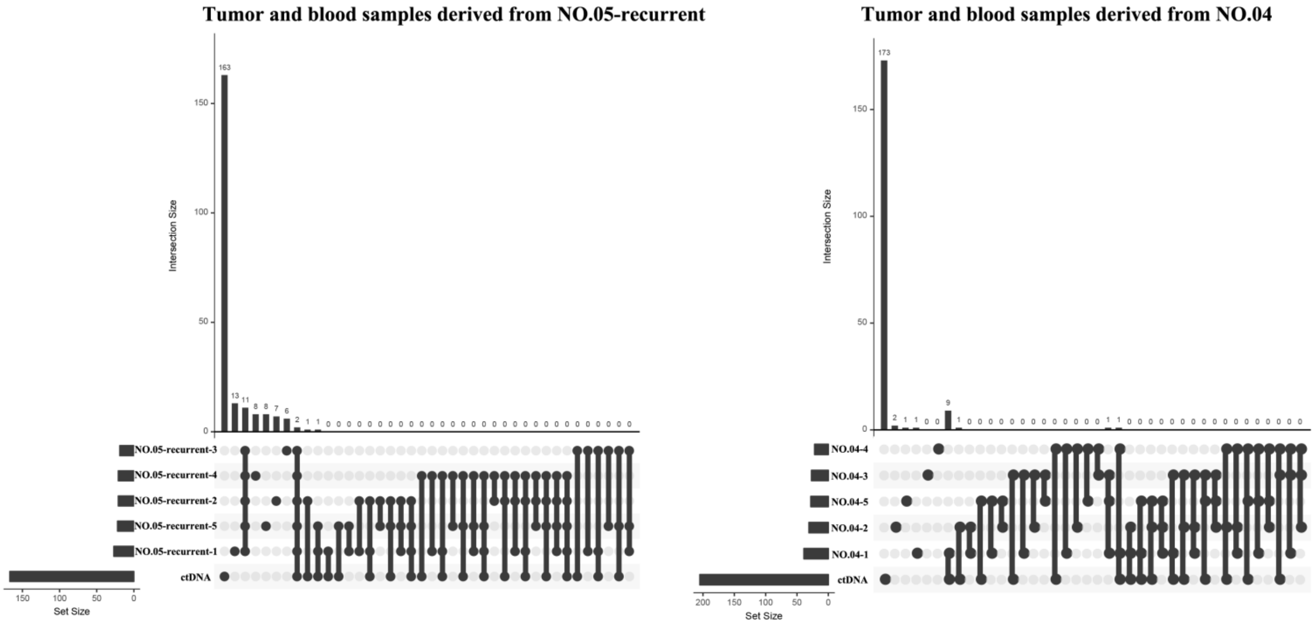

Supplementary Figure 6. Results of the ctDNA test and statistical analysis of the same mutations in the region of the ctDNA sequenced identified using exon sequencing at different tumor sites from patients NO. 05-recurrent and NO. 04.

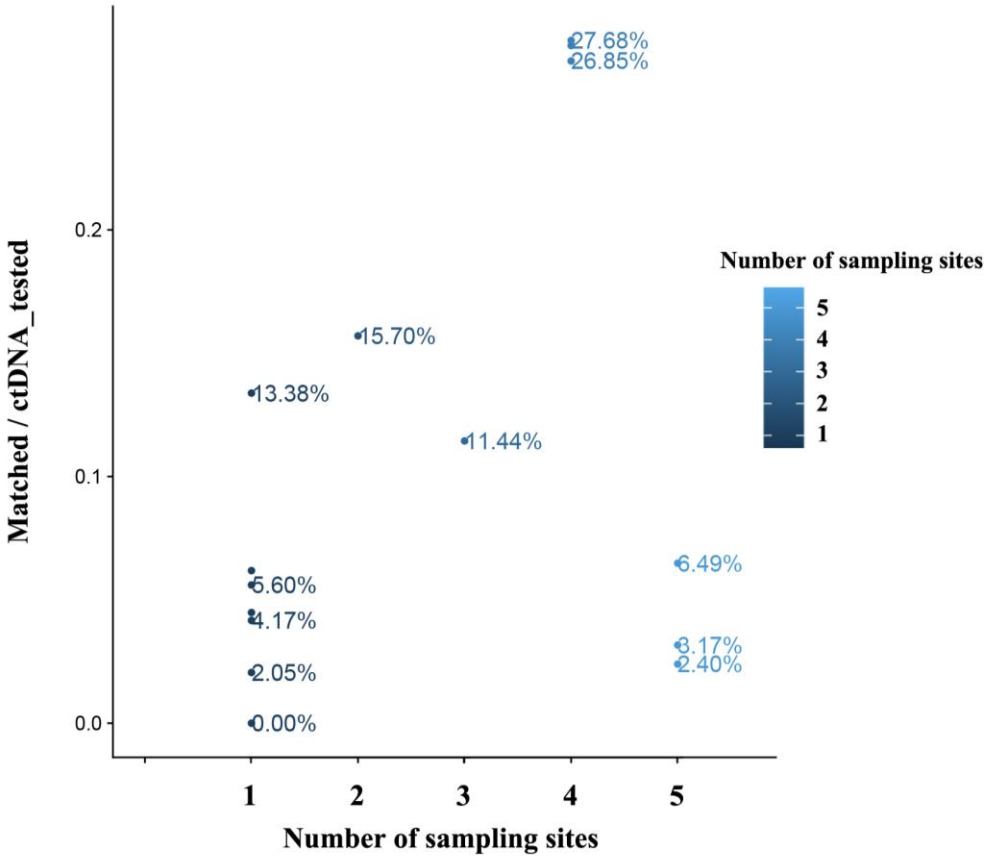

Supplementary Figure 7. Matching probability of ctDNA test and exon sequencing data in the region of the ctDNA sequenced from each patient.
