# Supplementary material for: Genomic landscapes by multiregion sequencing combined with circulation tumor DNA detection contribute to molecular diagnosis in glioblastomas
Source: Aging (Albany NY). 2019 Dec 10;11(23):11224–43. doi: 10.18632/aging.102526 (PMC6932900; doi:10.18632/aging.102526)
Supplement: Supplementary Table 1 [file aging-11-102526-s001..pdf]

SUPPLEMENTARY TABLE

Supplementary Table 1. Clinical data for the tumor samples

ID	Age	Gender	Sample type	Number of samples
NO.01	62	male	rGBM	3
NO.02	56	female	GBM	1
NO.03	51	female	GBM	1
NO.04	66	male	GBM	5
NO.05	45	male	GBM	4
NO.05-recurrent	46	male	rGBM	5
NO.06	19	male	GBM	1
NO.07	32	male	GBM	4
N0.08	62	male	GBM	1
N0.09	70	male	GBM	4
NO.10	61	male	GBM	1
NO.11	64	male	GBM	1
